# *Trem2* Splicing and Expression are Preserved in a Human Aβ-producing, Rat Knock-in Model of Trem2-R47H Alzheimer’s Risk Variant

**DOI:** 10.1038/s41598-020-60800-1

**Published:** 2020-03-05

**Authors:** Marc D. Tambini, Luciano D’Adamio

**Affiliations:** Department of Pharmacology, Physiology & Neuroscience New Jersey Medical School, Brain Health Institute, Jacqueline Krieger Klein Center in Alzheimer’s Disease and Neurodegeneration Research, Rutgers, The State University of New Jersey, 185 South Orange Ave, Newark, NJ 07103 USA

**Keywords:** Neuroscience, Alzheimer's disease

## Abstract

The R47H variant of the Triggering-Receptor-Expressed on Myeloid cells 2 (TREM2) increases the risk of Alzheimer’s disease (AD). Mutagenesis of exon 2 in Knock-in (KI) mouse models of the R47H variant introduced a cryptic splice site, leading to nonsense mediated decay. Since haploinsufficiency does not model Trem2-R47H function, a new rat KI model, the *Trem2*^*R47H*^ KI rat was created. Human Aβ has higher propensity to form toxic Aβ species, which are considered the main pathogenic entity in AD, as compared to rodent Aβ, the rat *Amyloid Precursor Protein* (*App*) gene was mutated to produce human Aβ. *Trem2* splicing and expression was measured in *Trem2*^*R47H*^ KI rat brains and microglia by qualitative and quantitative RT-PCR. Trem2 levels and Trem2 processing was assessed by Western analysis. APP metabolite levels were determined by enzyme-linked immunosorbent assay (ELISA), for Human Aβ and soluble APP, and Western analysis, for full length APP, βCTF and αCTF. *Trem2* expression and Trem2 levels are unchanged in *Trem2*^*R47H*^ KI rats. The artifactual splicing seen in KI mouse models is not present; additionally, two novel isoforms of rat *Trem2* are described. *Trem2*^*R47H*^ rat brains have lower human Aβ38, sAPPα and sAPPβ levels. Thus, *Trem2*^*R47H*^ KI rats may prove valuable to define pathogenic mechanisms triggered by the Trem2 R47H variant, including those mediated by toxic species of human Aβ peptides.

## Introduction

Alzheimer’s disease (AD) is a progressive neurodegenerative disorder and the most common form of dementia in the elderly^1^. AD is characterized by canonical histopathological lesions, which include extracellular Aβ plaques and intracellular tau tangles, as well synaptic deficits which result in cognitive impairment^2^. The evidence that microglia cells surround amyloid-plaques -both in AD patients^3^ and plaque-bearing mice^4^- and influence synaptic plasticity via synapse remodeling^5^ suggested a link between microglia and AD pathogenesis. Genetic evidence directly implicates microglia function in AD pathogenesis as genome-wide association studies have uncovered rare variants of *Triggering Receptor Expressed on Myeloid Cells 2* (*TREM2*), which was originally cloned in neutrophils and monocytes^6^ and whose expression in the central nervous system is restricted to microglia^7^, that increase the risk of developing AD^8^. *TREM2* is also expressed in osteoclasts and, in addition to modulating AD-risk, *TREM2* mutations cause frontotemporal dementia or Nasu-Hakola disease, a rare neurogenerative disorder with bone involvement and white matter loss^9^.

Multiple lines of evidence suggest a connection between TREM2 and Aβ plaques in mice. *Trem2* deletion reduces localization of microglia at Aβ plaques^10^, while TREM2 overexpression facilitates microglia-mediated clearance of Aβ-plaques^11^. Moreover, microglia isolated from *Trem2*-KO mice show reduced phagocytosis of lipoprotein-associated Aβ^12^. Trem2 has been found to bind Aβ directly, raising the possibility of Trem2 acting as a direct Aβ receptor^13^.

Because Aβ and Aβ-plaques are believed central to the pathogenesis of AD, it is postulated that *TREM2* mutations reduce TREM2 function and increase dementia risk by hampering the anti-amyloidogenic activity of microglia. The evidence that overexpression of dementia-associated variants *in vitro* shows deficits in cell surface trafficking of TREM2, in the case of p.T66M and p.Y38C, or ligand (lipids and Aβ)-binding, in the case of p.R47H and p.R62H^10^, support the hypothesis that disease-associated mutant TREM2 proteins are functionally deficient. To extend the mutational analyses to animal model organisms, several groups generated *Trem2*^*R47H*^ knock-in (KI) mice via CRISPR/Cas9^14–16^. Analysis of *Trem2* expression in these models revealed a reduction in *Trem2* levels that resulted from the generation of a cryptic splice site which introduces a premature stop codon^14,16^. This splicing impairment was not seen in transcriptional analysis of a human *TREM2-R47H* minigene, *TREM2-R47H* iPSC-derived human microglia-like cells, or in brain from patients heterozygous for the mutation^14^. Therefore, the *Trem2*^*R47H*^ KI mouse models more accurately reflect *Trem2* haploinsufficiency rather than the physiological effect of the R47H mutation on disease pathogenesis.

Here, we report the generation of a new *Trem2*^*R47H*^ KI rat model that faithfully replicates *Trem2* expression levels seen in wild-type rats. Together with the *Trem2* mutations we introduced mutations to “humanize” the rat Aβ sequence (*App*^*h*^ allele). Thus, *Trem2*^*R47H*^ KI rats produce human, and not rodent, Aβ from the endogenous rat *App* gene^17^. Rat *App* was humanized for the following reasons: 1) aggregated or oligomeric forms of Aβ are by and large considered the main pathogenic entity in AD; 2) human Aβ has higher propensity to form toxic Aβ species as compared to rodent Aβ; 3) as discussed above, *TREM2* pathogenic variants may facilitate neurodegeneration by increasing human Aβ-mediated neurotoxicity, Here, we characterize the effect of *Trem2*^*R47H*^ on human Aβ levels and APP processing. Our findings put forward a rat KI model of *Trem2* as a viable model for the investigation of p.R47H in animals producing human Aβ.

## Results

### Generation of *Trem2*^*R47H*^ rats carrying humanized *App* alleles (*App*^*h/h*^)

F0-*Trem2*^*R47H*^ rats were crossed to Long-Evans rats to generate F1- *Trem2*^*R47H/w*^ rats. These crossings were repeated four more times to obtain F5-*Trem2*^*R47H/w*^ rats. The probability that F5 rats carry unidentified off-target mutations (except those, if present, on Chr. 9) is ~1.5625%. To generate *Trem2*^*R47H*^ rats on a background in which rat *App* has a humanized Aβ region, F5-*Trem2*^*R47H/w*^ and *App*^*h/h*^ rats^17^ were crossed to generate F1-*Trem2*^*R47H/w*^; *App*^*h/w*^ rats. Progeny were crossed to remove the *App*^*w*^ allele. Henceforth, all *Trem2*^*R47H*^ rats have an *App*^*h/h*^ background and therefore produce human and not rodent Aβ species, and only these rats were used in all experiments.

To verify that the *Trem2*^*R47H*^ mutations were correctly inserted into *Trem2* exon-2, we amplified by PCR the *Trem2* gene exon-2 from *Trem2*^*w/w*^*, Trem2*^*R47H/w*^ and *Trem2*^*R47H*/*R47H*^ rats. Sequencing of the PCR products shows that the mutations were correctly inserted in the *Trem2*^*R47H/w*^ and *Trem2*^*R47H*/*R47H s*^ genomes (Fig. S1A).

### *Trem2* is correctly spliced in *Trem2*^*R47H*^ rats

**Figure 1:**
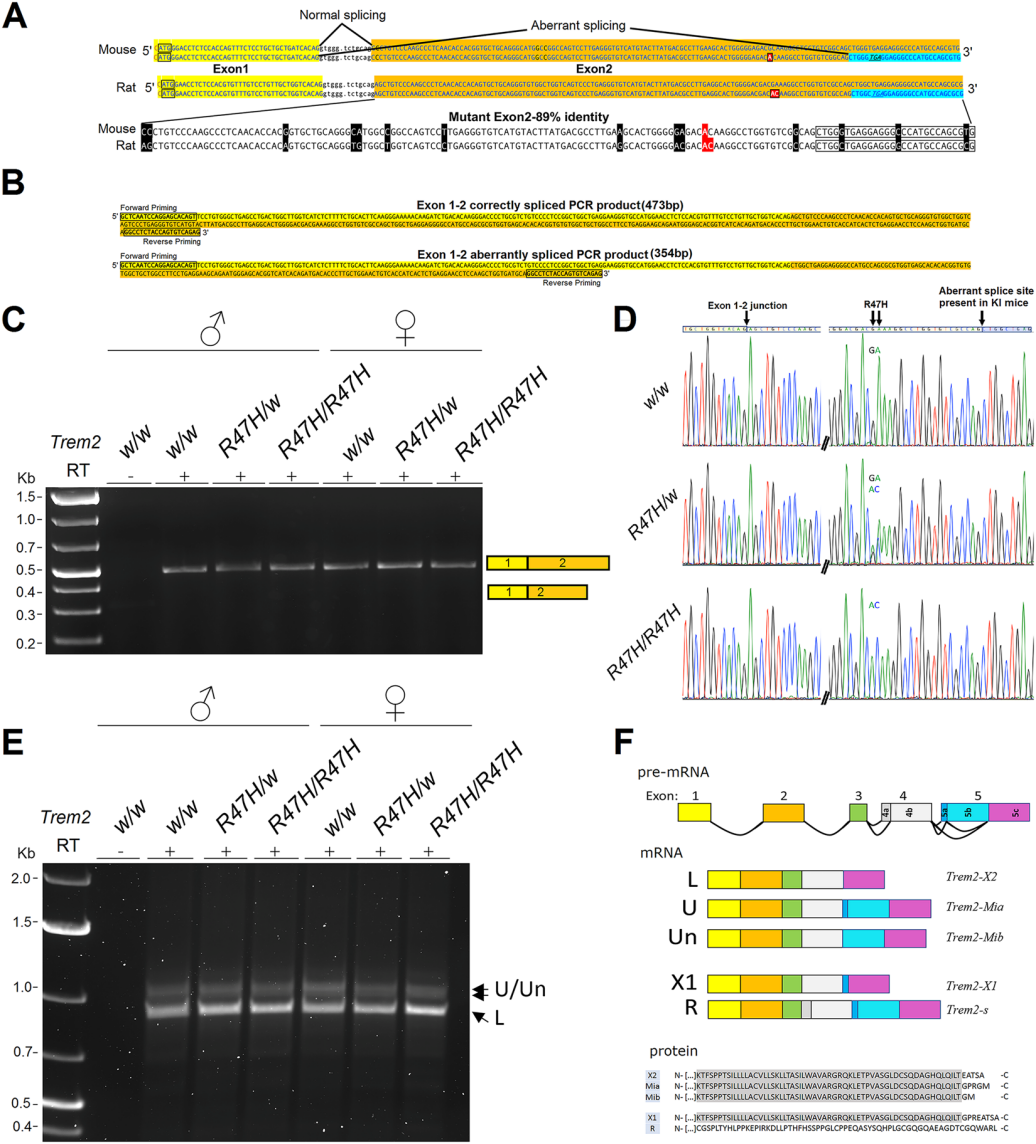
*Trem2* RNA splicing in *Trem2*^*R47H*^ rats. (**A**) Schematic of *Trem2* exon 1–2 junction in mouse and rat, WT (top sequence) and R47H mutant (bottom sequence). Exon 1 (yellow) normally splices to exon 2 (orange), however the R74H KI mutation (boxed red) causes the aberrant skipping of the 119 bp 5′ end of exon 2 and splicing to an internal site (cyan). This introduces a premature stop codon (underlined) which results in nonsense-mediated decay. Mutant R47H-containing exons 2 from mouse and rat share 89% identity (mismatches shaded). (**B**) Expected PCR products from normally and aberrantly spliced rat *Trem2*. Priming sites and sizes are indicated. (**C**) Semiquantitative RT-PCR of *Trem2* exon 1–2 junction. Total rat brain RNA from P20 male and female *Trem2*^*w/w*^, *Trem2*^*R47H/w*^, and *Trem2*^*R47H/R47H*^ rats was used to make cDNA. PCR amplification of *Trem2* exon 1–2 junction produced an amplicon consistent with the 473 bp size of the correctly spliced isoform. No other isoforms were visualized, and no amplification was present in the reverse transcriptase (RT) negative control. (**D**) Sanger sequencing of exon 1–2 PCR amplicons show no alterative splicing. R47H mutation is visible in *Trem2*^*R47H/w*^ and *Trem2*^*R47H/R47H*^ samples. (**E**) Semi-quantitative RT-PCR of coding sequences of *Trem2* mRNA. cDNA from rat brain was prepared as above. PCR amplification using 5′ and 3′-UTR priming produced three amplicons, two upper “U/Un” bands and one lower “L” band. (**F**) Gene organization of rat *Trem2* is shown. Sequencing shows that isoforms U and Un are novel isoforms (called *Trem2-Miα* and *Trem2-Miβ*) generated by alternative splicing of exon 5.

Exon 2 of rat and mouse *Trem2*^*R47H*^ share 89% similarity (Fig. 1A). To determine if the alternative splicing event that is responsible for the loss of *Trem2* expression in KI mice is also present in KI rats, *Trem2* splicing was tested in *Trem2*^*w/w*^, *Trem2*^*R47H/w*^, and *Trem2*^*R47H/R47H*^ rats. Alternative splicing of exon 2 in *Trem2*^*R47H*^ mice results a splice variant that lacks 119 bp of the 5′ end of exon 2^14^. Semiquantitative RT-PCR, using primers that flank this splice site failed to detect such an isoform in *Trem2*^*R47H*^ rat brain cDNA (Fig. 1B,C). Correctly spliced *Trem2* was confirmed by cDNA sequencing, with the R47H mutation apparent in *Trem2*^*R47H/w*^ and *Trem2*^*R47H*/*R47H*^ rats (Fig. 1D). To test if the R47H mutation is responsible for alterative splicing events in other regions of the gene, rat *Trem2* cDNA was amplified with primers in the 5′ and 3′ untranslated regions. Three isoforms were present in *Trem2*^*w/w*^, *Trem2*^*R47H/w*^, and *Trem2*^*R47H/R47H*^ rats, with no apparent qualitative differences between the genotypes (Fig. 1E). Sequencing of the cDNAs revealed one known, *Trem2-X2* (RefSeq Accession number: XM_006244425.3), which is identical to our “L” or “lower” isoform, and two novel *Trem2* isoforms, herein named *Trem2-Miα* (Gene Bank Accession number: MN207145) and *Trem2-Miβ* (Gene Bank Accession number: MN207146). The exon composition of all known *Trem2* isoforms is shown in Fig. 1F (schematic) and Fig. S1B (full sequence). The cDNA sequence of all known *Trem2* isoforms is shown in Fig. S1D. The predicted amino acid sequence of the 3 isoforms expressed in the central nervous system differs with respect to the extreme C-terminus (Fig. 1G).

### *Trem2* is expressed at normal levels in *Trem2*^*R47H*^ rats

**Figure 2:**
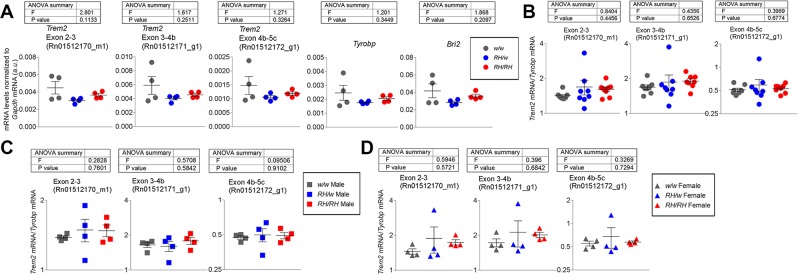
*Trem2* RNA expression in *Trem2*^*R47H*^ rats. (**A**) Levels of *Trem2* mRNA, normalized to *Gapdh* mRNA, from brain lysate were measured in *Trem2*^*w/w*^, *Trem2*^*R47H/w*^, and *Trem2*^*R47H/R47H*^ rats by quantitative RT-PCR. No significant differences between *Trem2*^*w/w*^, *Trem2*^*R47H/w*^, and *Trem2*^*R47H/R47H*^ rats were evident with any of the 3 probes tested (exon junctions indicated). Levels of *Tyrobp* and *Bri2* mRNA, normalized to *Gapdh* mRNA, from brain lysate were also unchanged by *Trem2*^*R47H*^. N = 4 P20 rats (2 males, 2 females) per genotype. (**B**) Levels of *Trem2* mRNA, normalized to *Tyrobp* RNA, from bead-isolated microglia showed no significant differences in *Trem2*^*w/w*^, *Trem2*^*R47H/w*^, and *Trem2*^*R47H/R47H*^ rats with any of the 3 probes tested. N = 8 P28 rats (4 males, 4 females) per genotype. (**C**) Segregation of values from male rats and (**D**) female rats from the data in panel B show no sex-dependent effects in the unchanged *Trem2* mRNA levels in *Trem2*^*R47H*^ rats. N = 4 P28 rats (4 males, or 4 females, respectively) per genotype. Data are represented as mean ± SEM and were analyzed by ordinary one-way ANOVA.

Mis-splicing of *Trem2* in KI mice results in decreased *Trem2* expression, via nonsense mediated decay^14^. *Trem2* expression in *Trem2*^*w/w*^, *Trem2*^*R47H/w*^, and *Trem2*^*R47H/R47H*^ rat brains was determined by quantitative RT-PCR. Three *Trem2* probes (two probes, Rn01512170_m1 and Rn01512171_g1, detect isoforms X2, Miα, and Miβ, and one probe, Rn01512172_g1, only detects isoform X2) were used. No differences between the genotypes were seen in *Trem2* expression with any probe used, and similarly no differences were evident in the microglial gene *Tyrobp*^18^ and *Bri2*, a gene that modulates APP function/metabolism^19–24^, Aβ levels and whose mutations cause familial dementia similar to familial AD^25–30^ (Fig. 2A). The expression levels were normalized to expression of the housekeeping gene *Gapdh*. These analyses indicate that R47H mutation does not affect *Trem2* splicing or RNA expression in *Trem2*^*R47H*^ KI rat brains.

A larger cohort of rats was generated, and microglia were isolated from rat brains to test for sex-specific differences in *Trem2* expression in microglia. Purity of the cell population was confirmed by flow cytometry (Fig. S2A). Given the changes in microglial gene expression caused by culturing^31^, RNA was extracted immediately upon microglia purification. *Trem2* expression was tested by RT-PCR with the 3 probes as above in male and female *Trem2*^*w/w*^, *Trem2*^*R47H/w*^, and *Trem2*^*R47H/R47H*^ microglia and normalized to *Tyrobp* expression. Similar to brain levels of *Trem2*, microglia levels of *Trem2* do not significantly differ between *Trem2*^*w/w*^, *Trem2*^*R47H/w*^, and *Trem2*^*R47H/R47H*^ rats, either as a group (Fig. 2B) or separated by sex (Fig. 2C,D). *Tyrobp* levels were chosen for normalization as this was the method of analysis performed in *Trem2*^*R47H*^ KI mice^14^, where *Tyrobp* levels do not vary in relation to *Trem2*. In *Trem2*^*R47H*^ KI rats, the assumption is made, but not formally tested, that *Tyrobp* levels also do not vary. Nevertheless, since only microglia cells express *Trem2* in the brain, the data in Fig. 2A would be sufficient to conclude that the R47H KI mutation does not affect the splicing nor abundance of any *Trem2* transcript detected.

### Trem2 protein levels and processing are normal in *Trem2*^*R47H*^ rats

**Figure 3:**
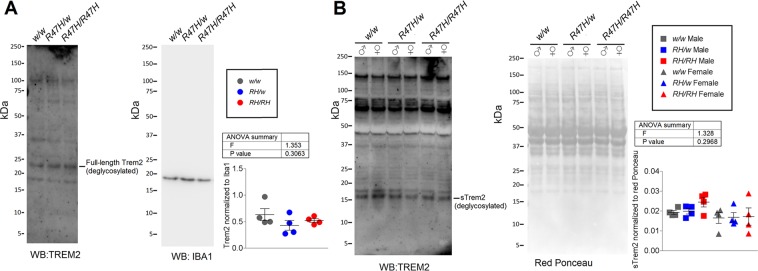
Trem2 and sTrem2 protein levels in *Trem2*^*R47H*^ rats. (**A**) Levels of Trem2 were determined from deglycosylated total microglia protein from *Trem2*^*w/w*^, *Trem2*^*R47H/w*^, and *Trem2*^*R47H/R47H*^ rat brains by Western analysis. Trem2 values were normalized to Iba1. N = 12 P1.5–2-month-old rats (2 males, 2 females for each genotype). (**B**) Levels of sTrem2 were determined from deglycosylated soluble rat brain fractions from *Trem2*^*w/w*^, *Trem2*^*R47H/w*^, and *Trem2*^*R47H/R47H*^ rats by Western analysis. sTrem2 values were normalized to red Ponceau intensity. N = 8 P28 rats (4 males, 4 females for each genotype). Data are represented as mean ± SEM and were analyzed by ordinary one-way ANOVA.

Because Trem2 is glycosylated, therefore giving a signal greatly dishomogeneous in size, and its expression is restricted to microglia, to detect Trem2 protein levels in a quantitative manner by Western analysis, it was necessary to first extract microglia from rat brains, and then to deglycosylate total microglial proteins. Peripheral myeloid cells were removed from brain tissue via intracardiac catheterization and perfusion. Brain tissue was enzymatically and mechanically dissociated into a single cell suspension, which was then used as the input for the positive selection of CD11b/c expressing cells. Since Iba1 is enriched, we hereafter refer to this fraction as “microglia,” but it remains possible other CD11b/c expressing cells may also be present (Fig. S2B). Trem2 antibody recognizes a glycosylated band present in microglia and absent in non-microglial cell types isolated from rat brain (Fig. S2B). Microglia isolated from *Trem2*^*w/w*^, *Trem2*^*R47H/w*^, and *Trem2*^*R47H/R47H*^ rat brains show no significant differences in Trem2 content (Fig. 3A). Trem2 is processed^32^ at the cell surface by A Disintegrin and Metalloproteinase 10 (ADAM10)^33^ to release a soluble N-terminal ectodomain (sTrem2). As shown in Fig. 1G, sTrem2 molecules produced by the X1, Miα and Miβ isoforms are identical. sTrem2 is present in soluble fractions of total rat brain lysate and, when deglycosylated, migrates as a ~16 kDa band that is also detected in media conditioned by primary microglia and Trem2-expressing HEK cells (Fig. S2C). sTrem2 content of soluble brain lysate was determined by Western analysis of *Trem2*^*w/w*^, *Trem2*^*R47H/w*^, and *Trem2*^*R47H/R47H*^ rat brain S100 fractions, and no significant difference was seen across genotypes in a sex-independent manner (Fig. 3B).

### Subtle changes in Aβ and soluble APPs in *Trem2*^*R47H*^ rats

TREM2 modulates the microglial response to plaques; therefore, APP processing and Aβ clearance may be affected by the R47H mutation. *Trem2*^*R47H*^ rats were generated on an *App*^*h/h*^ background, which as of 3 months of age, does not exhibit plaque pathology^17^, thereby allowing analysis of soluble monomeric or oligomeric forms of human Aβ, which interact directly with TREM2 -an interaction that is reduced in R47H mutants^13^ - without the confounding effect of human Aβ aggregation. Thus, we determined the steady-state levels of soluble human Aβ species. Full length APP, soluble APPs (sAPPα/sAPPβ), and APP C-terminal fragments (αCTF/βCTF) were also measured to correlate any changes seen in Aβ levels with alterations in APP abundance or processing.

**Figure 4:**
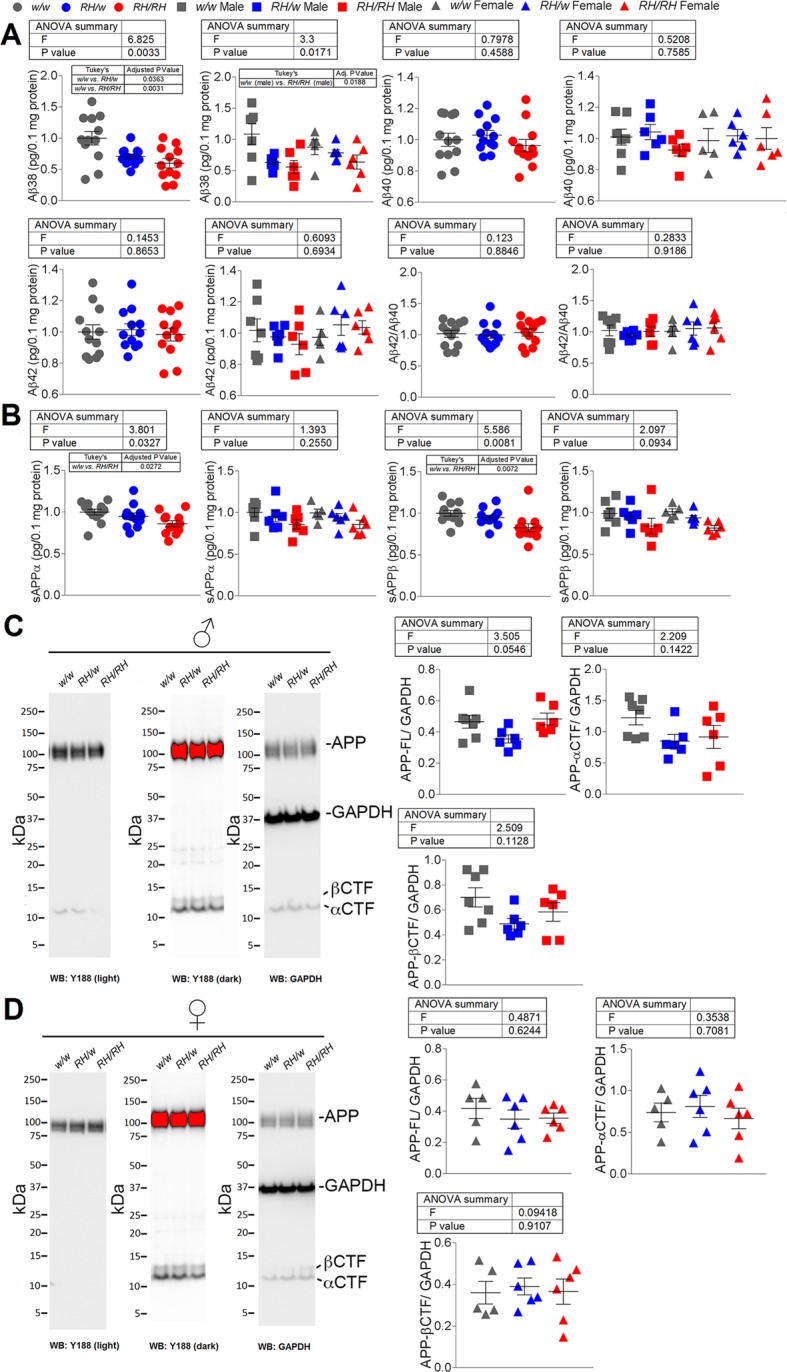
APP metabolite levels in *Trem2*^*R47H*^ rats. (**A**) Levels of Aβ38, Aβ40, and Aβ42 were determined by ELISA of brain lysate of *Trem2*^*w/w*^, *Trem2*^*R47H/w*^, and *Trem2*^*R47H/R47H*^ rats. Data from total animals per genotype and from each sex are presented. Values are presented as normalized to *Trem2*^*w/w*^ values. N = 12 P28 rats (7 males, 5 females for *Trem2*^*w/w*^ and 6 males, 6 females for *Trem2*^*R47H/w*^ and *Trem2*^*R47H/R47H*^). (**B**) Levels of sAPPα and sAPPβ were determined by ELISA of brain lysate in *Trem2*^*w/w*^, *Trem2*^*R47H/w*^, and *Trem2*^*R47H/R47H*^ rats. Data from total animals per genotype and from each sex are presented. Values are presented as normalized to *Trem2*^*w/w*^ values. N = 12 P28 rats (7 males, 5 females for *Trem2*^*w/w*^ and 6 males, 6 females for *Trem2*^*R47H/w*^ and *Trem2*^*R47H/R47H*^). (**C**) Levels of APP metabolites, i.e. full-length APP, αCTF, and βCTF, were determined by Western analysis of brain lysate of *Trem2*^*w/w*^, *Trem2*^*R47H/w*^, and *Trem2*^*R47H/R47H*^ male rats. Quantitation of Western blots are on the right. Signal intensity of APP metabolites were normalized to GAPDH levels. N = 6 or 7 P28 rats (7 males for *Trem2*^*w/w*^ and 6 males for *Trem2*^*R47H/w*^ and *Trem2*^*R47H/R47H*^). (D) Levels of APP metabolites were determined by Western analysis of brain lysate of *Trem2*^*w/w*^, *Trem2*^*R47H/w*^, and *Trem2*^*R47H/R47H*^ female rats. Quantitation of Western blots are on the right. Signal intensity of APP metabolites were normalized to GAPDH levels. N = 5 or 6 P28 rats (5 females for *Trem2*^*w/w*^ and 6 females for *Trem2*^*R47H/w*^ and *Trem2*^*R47H/R47H*^). Data are represented as mean ± SEM. Data were analyzed by ordinary one-way ANOVA followed by post-hoc Tukey’s multiple comparisons test when ANOVA showed statistically significant differences.

Levels of human Aβ species (Aβ38, Aβ40, and Aβ42) and soluble APP (sAPPα and sAPPβ) in total brain lysates were detected by human Aβ specific-ELISA. No differences were seen in Aβ40 or Aβ42 levels in *Trem2*^*w/w*^, *Trem2*^*R47H/w*^, and *Trem2*^*R47H/R47H*^ rats in a sex-independent manner (Fig. 4A), nor was the Aβ42/Aβ40 ratio altered (Fig. 4A). Notably, human Aβ38 levels were significantly decreased in *Trem2*^*R47H/w*^ and *Trem2*^*R47H/R47H*^ compared to *Trem2*^*w/w*^ rats, with significance seen in males and a trend in females (Fig. 4A). Small but statistically significant decreases in both sAPPα and sAPPβ were evident in *Trem2*^*R47H/R47H*^ as compared to *Trem2*^*w/w*^, while higher power is needed to see if the significant differences are maintained when segregating by sex (Fig. 4B). Levels of full-length APP and APP-CTFs were detected by Western analysis and found to be unchanged in *Trem2*^*w/w*^, *Trem2*^*R47H/w*^, and *Trem2*^*R47H/R47H*^ male and female rats (Fig. 4C,D).

Overall, these data indicate that soluble human Aβ levels are normal, with the exception of lower levels of Aβ38 in *Trem2*^*R47H/w*^ and *Trem2*^*R47H/R47H*^ rats. The concurrent decrease of sAPPs in *Trem2*^*R47H/R47H*^ rats would suggest that the cause of the Aβ38 decrease is a reduction of APP processing. Indeed, sAPPα and sAPPβ are a better indicator of α- and β-processing of APP, respectively, than the corresponding CTFs, which undergo further metabolism via multiple pathways (e.g. γ-secretase, caspase, autophagosomal/lysosomal degradation^34–37^). Thus, changes in Trem2 function may affect a discrete pool of βCTFs that are preferentially processed to position 38. Reduction in steady-state levels of Aβ38 may also be caused by a shorter half-life of Aβ38 as compared to Aβ40 and Aβ42 in rats carrying the *Trem2*^*R47H*^ mutation, suggesting that WT Trem2 may more efficiently bind and clear longer Aβ as compared to shorter Aβ species. In addition, the *Trem2*^*R47H*^ mutation may also reduce the half-life of sAPPα and sAPPβ. These possibilities do not need to be mutually exclusive.

## Discussion

Although some TREM2 mutations result in haploinsufficiency via decreased cell surface trafficking^33^, the TREM2-R47H mutant is present in normal levels at the cell surface^33^ and in human brain^38^. Several groups have recently created KI mouse models of the R47H mutation and found that mutagenesis of exon 2 introduces a cryptic splice site^14,16^. This results in the production of isoforms that use a premature stop codon and are targeted for nonsense mediated decay. *Trem2*^*R47H*^ KI mice are haploinsufficient, and therefore not suited to model the mutation.

We have generated and validated a KI rat model of the R47H mutation that shows preserved *Trem2* splicing and unchanged levels of Trem2 expression. Mouse and rat *Trem2* exon2 sequences differ by 11%, and it may be this dissimilarity that underlies the absence of a cryptic splice site in *Trem2*^*R47H*^ KI rats. Additionally, we report two novel isoforms of rat *Trem2* that differ with respect to their C-termini. Future experiments are needed to characterize the different functions of these isoforms.

The generation of *Trem2*^*R47H*^ KI rat on *App*^*h/h*^ KI background ensures the production of only human and not rodent Aβ and thus has the additional advantage of being well suited for the study of APP, APP metabolism and human Aβ toxicity which may not be replicated by rodent Aβ. The use of knock-in APP rats rather than more common transgenic APP models avoids several confounding factors: overexpression of APP above physiological levels, the disruption of genes in the transgene integration sites, and the use of exogenous promoters which do not replicate the temporal, cell type-specific or spatial expression of the endogenous gene. Using this approach, we detected a significant decrease in soluble APPs and Aβ38 in *Trem2*^*R47H*^ rats. While the physiological function of Aβ38 is unknown, there is some evidence that short forms of Aβ, including Aβ38, attenuate toxicity of longer Aβ species^39^, thus the decrease in Aβ38 in *Trem2*^*R47H*^ rats may be of pathogenic importance.

## Methods

### Rats and ethics statement

Rats were handled according to the Ethical Guidelines for Treatment of Laboratory Animals of the NIH. The procedures were described and approved by the Institutional Animal Care and Use Committee (IACUC) at Rutgers.

### Generation of Trem2 KI rats

Generation of rats carrying the Trem2 gene with the R47H mutation on a background with rat App containing a humanized Aβ region. The rat Trem2 gene (GenBank accession number: NM_001106884.1; Ensembl: ENSRNOG00000013578) is located on rat chromosome 9. We created Long-Evans rats with point mutation CGA > CAC at rat Trem2 locus by CRISPR/Cas-mediated genome editing. These mutations will create a rat that carries a Trem2 gene coding for rat Trem2 R47H variant. The rat Trem2 gene is comprised of 5 exons, with the ATG start codon in exon 1 and TAA stop codon in exon 4; the CGA codon is located in exon 2. Thus, exon 2 was selected as target site. gRNA targeting vector and oligo donor (with targeting sequence, flanked by 120 bp homologous sequences combined on both sides) was designed as follows.

Cas9 RNA, gRNA generated by *in vitro* transcription and oligo donor were co-injected into zygotes for production of rats carrying these knock-in (KI) mutations by homology-directed repair. To verify CRISPR-induced mutation the pups were genotyped by PCR, followed by sequence analysis. The rat *Trem2* locus was amplified by PCR with the following specific forward (F) and reverse (R) primers: F- ATATAGTTTGCTGCTCCTGGTAGACGC; R- AAAGTCACACAACAATGAGACCTGGC.

Cas9 RNA, sgRNA and oligo donor are co-injected into zygotes, but homology-directed repair can occur even after few cell cycles. Thus, injected rats can have a mixture of correctly targeted alleles and alleles carrying aberrant mutations or no mutations. To identify rats carrying correctly targeted *Trem2* alleles, the PCR products were cloned into TA vectors and 10 clones were sequenced using forward primer: 5-CAAAGGTGCAACCAGCCAGTG-3′. This analysis showed that RatID#10 had two types of alleles:

Thus, RatID#10 was identified as a positive chimeric founder F0-*Trem2*^*R47H*^ rat. The unintended *Trem2*-*δ*18 allele was removed in subsequent crosses.

*Off-target analysis for gRNA*. Homology-directed repair can cause off-target mutations in genetic sites that have high homology with the gRNAs. We identified potential off-target sites for our gRNA. Based on this analysis, RatID#10 (F0-*Trem2*^*R47H*^ rat) has been analyzed for mutations in these most likely off-target mutation sites. Mismatched bases are in red.

*Off-target analysis of targeting sequence gRNA: GAGGCACTGGGGACGACGAAAGG*. Five potential off-target sites have been identified (mismatched bases with the targeting sequence are in red.) These sites have been amplified by PCR and sequenced.

### Rat brain preparation

Rats were anesthetized with isoflurane and perfused via intracardiac catheterization with ice-cold PBS. Brains were extracted and homogenized using a glass-teflon homogenizer (w/v = 100 mg tissue/1 ml buffer) in 250 mM Sucrose, 20 mM Tris-base pH 7.4, 1 mM EDTA, 1 mM EGTA plus protease and phosphatase inhibitors (ThermoScientific), with all steps carried out on ice or at 4 °C. Total lysate was solubilized with 0.1% SDS and 1% NP-40 for 30 min rotating. Solubilized lysate was spun at 20,000 g for 10 m, the supernatant was collected and analyzed by ELISA and Western blotting. For analysis of soluble Trem2, brain lysate was spun at 100,000 g for 30 min, and supernatant (S100) was collected for further analysis.

### Microglia isolation

Rats were perfused with PBS, and total brain was extracted. Brains were enzymatically and mechanically dissociated into a cell suspension using the Adult Brain Dissociation Kit and gentleMACS Octo Dissociator (Miltenyi). Microglia were isolated using CD11b/c magnetic microbeads (Miltenyi) according to the manufacturer’s instructions. Microglia were used immediately for RNA and protein extraction. For validation of microglia purity, microglia were also plated in microglia media (1X MEM, 4% Fetal Bovine Serum, 6% Horse Serum, 0.6% glucose, 1 mM sodium pyruvate, 1mM L-glutamine, and 1% pen/strep) in 37 °C and 5% CO2. Purity was confirmed with FACS analysis of CD11b and CD45 expression with CD11b-FITC and CD45-APC-Vio770 respectively (Miltenyi). Data supporting the purity of the microglia isolation are contained in the Supplemental Fig. S2.

### Quantitative and semi-quantitative RT-PCR

Total brain RNA or microglia RNA was extracted with RNeasy RNA Isolation kit (Qiagen) and used to generate cDNA with a High-Capacity cDNA Reverse Transcription Kit (Thermo) with oligo dT priming. 50 ng cDNA, TaqMan™ Fast Advanced Master Mix (Thermo 4444556), and the appropriate TaqMan (Thermo) probes were used in the real time polymerase chain reaction. Samples were analyzed on a QuantStudio 6 Flex Real-Time PCR System (Thermo), and relative RNA amounts were quantified using LinRegPCR software (hartfaalcentrum.nl). The probes Rn01512170_m1 (exon junction 2–3), Rn01512171_g1 (exon junction 3–4) and Rn01512172_g1 (exon junction 4–5) was used to detect rat *Trem2*. *Tyrobp* was detected with Rn01475740_m1 and *Bri2* was detected with Rn01468316_mH.

For semiquantitative analysis of *Trem2* splicing, 2 µl cDNA was used in the following PCRs. To test *Trem2* exon 1–2 splicing, forward primer 5-GCTCAATCCAGGAGCACAGT-3 and reverse primer 5-CTCTGACACTGGTAGAGGCC-3 were used, and cycling conditions were as follows: 95 °C 1 min; (98 °C 10s, 57 °C 15s, 72 °C 30s) x35 cycles; 72 °C 10 min. To test splicing of the entire *Trem2* gene, a nested PCR approach with primers in the 5′ and 3′UTR was used. The first PCR used forward primer 5-TAGTCCTGGCTGTTGGTTGC-3 and reverse primer 5-ACAGACGTTTACCAGCAACC-3, and cycling conditions were as follows: 95 °C 1 min; (98 °C 30s, 57 °C 15s, 72 °C 1 min) × 10 cycles; 72 °C 10 min. 1 µl of this PCR was used as substrate for further amplification with forward primer 5-TCAATCCAGGAGCACAGTTCC-3 and 5-CCACTCAACGCAGATGCAGC-3, and cycling conditions were as follows: 95 °C 1 min; (98 °C 30s, 62 °C 15s, 72 °C 1 min) × 25 cycles; 72 °C 10 min. PCR products were separated on 1% agarose gel, stained with ethidium bromide, and visualized on a ChemiDoc (Biorad). Gel bands were excised, and cDNA was recovered with a DNA extraction Kit (Qiagen) and sequenced with both reverse and forward PCR primers.

### Western analysis

Protein content was quantified by Bradford analysis prior to solublization. 15 µg of protein was brought to 15 µl with PBS and LDS Sample buffer-10% β-mercaptoethanol (Invitrogen NP0007) to 1X and loaded on a 4–12% Bis-Tris polyacrylamide gel (Biorad 3450125). Proteins were transferred onto nitrocellulose at 25 V for 7 min using the Trans-blot Turbo system (Biorad) and visualized by red Ponceau staining. Membranes were blocked 30 min in 5%-milk (Biorad 1706404), washed extensively in PBS/Tween20–0.05%, and primary antibody was applied overnight at 4 °C, at 1:1000 dilution in blocking solution (Thermo 37573). The following antibodies were used: Y188 (APP-C-terminus, Abcam ab32136), Trem2-2B5 (N-terminus, Novus NBP1-07101), Iba1 (Wako 019-19741), and GAPDH (Sigma G9545). Anti-sheep (Novus, NBP1-73267) and a 1:1 mix of anti-rabbit (Southern Biotech, OB405005) and anti-rabbit (Cell Signaling, 7074), were diluted 1:1000 in 5% milk and used against sheep and rabbit primary antibodies for 30 min, RT, with shaking. Blots were developed with West Dura ECL reagent (Thermo, PI34076) and visualized on a ChemiDoc MP Imaging System (Biorad). Signal intensity was quantified with Image Lab software (Biorad). Data were analyzed using Prism software and represented as mean ± SEM.

Prior to Western analysis, samples for Trem2 and soluble Trem2 protein quantitation required deglycosylation to yield a single discrete band for accurate quantitation. For deglycosylation experiments, total brain lysate or total microglia was solubilized with 1% NP-40 for 30 min rotating, spun at 20,000 g and the supernatant was used as input for deglycosylation reactions, according to the manufacturer’s specifications (NEB P6044S). S100 soluble brain fractions and conditioned media were also deglycosylated directly, with no prior solubilization step.

### ELISA

For analysis of Aβ and sAPPs, the following Meso Scale Discovery kits were used: Aβ38, Aβ40, and Aβ42 were measured with V-PLEX Plus Aβ Peptide Panel 1 6E10 (K15200G) and V-PLEX Plus Aβ Peptide Panel 1 and sAPPα/β were measured with sAPPα/sAPPβ (K15120E), according to the manufacturer’s recommendations. Plates were read on a MESO QuickPlex SQ 120. Data were analyzed using Prism software and represented as mean ± SEM.

### *Trem2* cDNA plasmid generation

Total brain cDNA was amplified with 5′-GCCGGATCCGCCACCATGGAACCTCTCCACGTGTTTGTCC-3′ and 5′- GGCGCGGCCGCCCACTCAACGCAGATGCAGC-3′ primers and cloned into pcDNA3.1+ using a BamHI/NotI cloning strategy. Clones were analyzed by Sanger sequencing and found to either contain cDNA that codes for *Trem2-X2* (RefSeq Accession number: XM_006244425.3) or *Trem2-Miα* (Gene Bank Accession number: MN207145). The latter construct was transfected into HEK cells and lysate/media was analyzed for the experiment shown in Fig. S2.

### Statistical analysis

Statistical significance was evaluated using Ordinary one-way ANOVA followed by Post-hoc Tukey’s multiple comparisons test when applicable (i.e. when the Ordinary one-way ANOVA test showed statistical significance). Statistical analysis was performed with GraphPad Prism v8 for Mac. Significant differences were accepted at p < 0.05.

## Supplementary information

Supplementary Dataset 1.
