# Supplementary material for: Trem2 Splicing and Expression are Preserved in a Human Aβ-producing, Rat Knock-in Model of Trem2-R47H Alzheimer’s Risk Variant
Source: Sci Rep. 2020 Mar 5;10:4122. doi: 10.1038/s41598-020-60800-1 (PMC7058057; doi:10.1038/s41598-020-60800-1)
Supplement: Supplementary file 1 — Supplementary Dataset 1. [file 41598_2020_60800_MOESM1_ESM.docx]

***Trem2* Splicing and Expression are Preserved in a Human Aβ-producing, Rat Knock-in Model of Trem2-R47H Alzheimer’s Risk Variant**

Marc D. Tambini & Luciano D’Adamio*

Department of Pharmacology, Physiology & Neuroscience New Jersey Medical School, Brain Health Institute, Jacqueline Krieger Klein Center in Alzheimer's Disease and Neurodegeneration Research, Rutgers, The State University of New Jersey, 185 South Orange Ave, Newark, NJ, 07103, USA.

*corresponding author, email: luciano.dadamio@rutgers.edu

**Supplementary information**

**
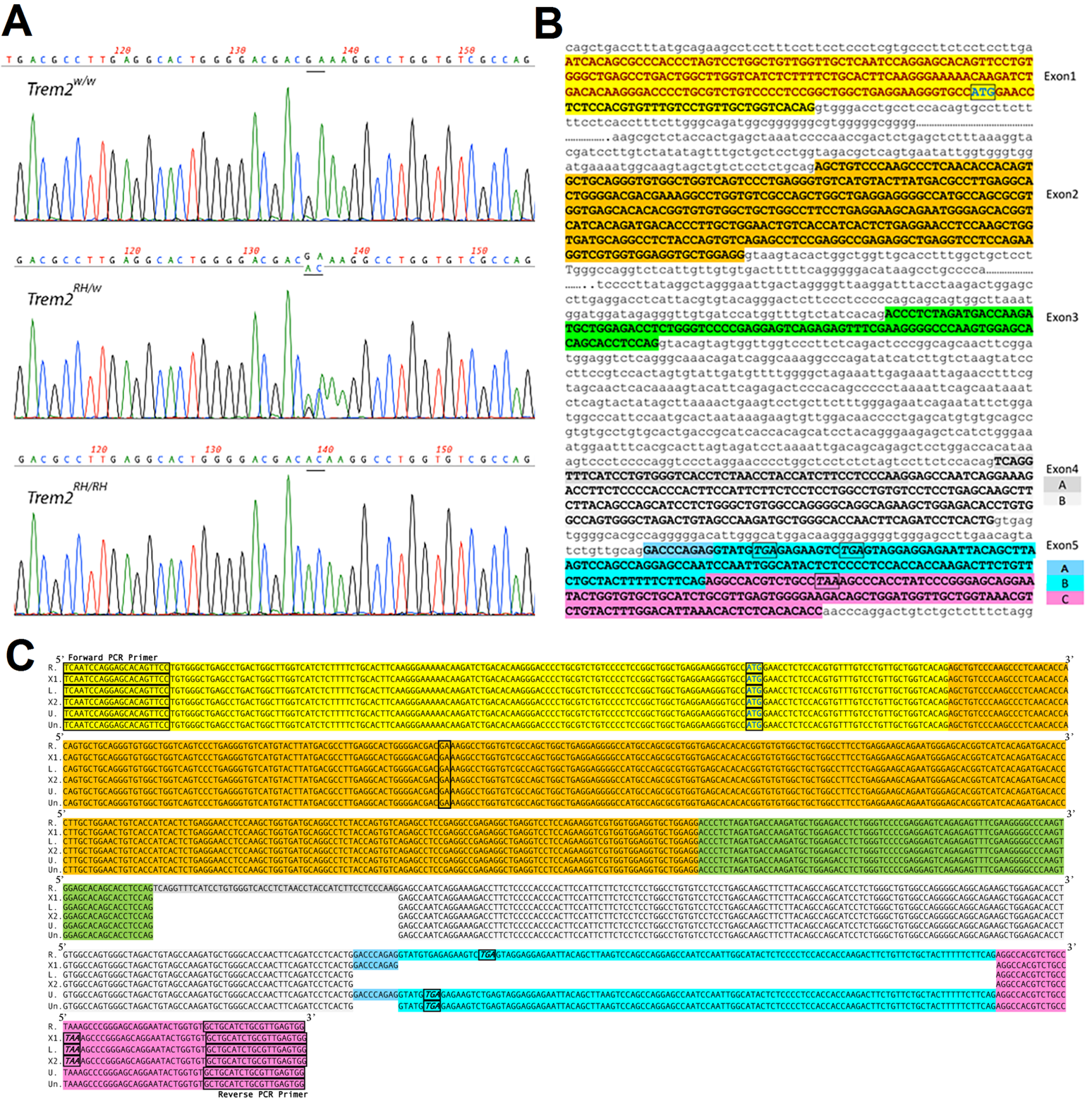
Figure S1.** ***Trem2* reference sequences.** (A) Sanger sequencing of PCR product from genomic DNA of *Trem2^w/w^*, *Trem2^R47H/w^*, and *Trem2^R47H/R47H^* rats. Pathogenic mutations are underlined. (B) *Trem2* gene organization, including the new splicing sites that are used by isoforms *Trem2-Miα* and *Trem2-Miβ*. Each exon is labeled and intronic sequences are abbreviated by ellipses. Start codon and stop codons for each isoform are boxed. (C) *Trem2* cDNA sequences for isoforms R, X1, L, X2, U (called *Trem2-Miα),* and Un (called *Trem2-Miβ).* Start codon and stop codons for each isoform are boxed. Forward and reverse primers used in Fig. 1 are indicated. Wild-type DNA is shown, but the two mutated bases that define *Trem2^R47H^* in Exon 2 are boxed.

**

Figure S2.** **Validation of microglia preparation and Trem2 antibody for the detection of full length and soluble Trem2.** (A) FACS analysis of stages of microglia preparation. All cells were double stained for microglia markers CD45 and CD11b/c. Upper left panel shows labeling of single cell suspension from total rat brain, post myelin removal. Upper right shows label of flow through fraction collected after single cell suspension was passed through CD11b/c microbeads. Lower left shows eluate from CD11b/c microbeads. Lower right panel has the cells collected from elution after 2 days in cell culture. Note that CD11b/c positivity results after two days of cell culture is likely the result of the blockage of the CD11b/c epitope by the CD11b/c microbeads during isolation, which resolves after cell culturing. (B) Western analysis of microglia for Trem2 content. Flow through (FT) and microglia eluate (MG) from CD11b/c microbeads were deglycosylated and stained with an antibody that recognizes the N-terminus of Trem2. Trem2 signal shown here could be an underestimation of total Trem2 signal, as the enzymatic dissociation of total brain could degrade a fraction of cell surface Trem2. (C) Western analysis of full length Trem2 in Trem2-transfected HEK cells (left). Western analysis of soluble Trem2 (sTrem2) from Trem2-transfected HEK cells, soluble fractions of total rat brain, and media conditioned by primary microglia (middle and right). Media from Trem2-transfected HEK cells shows a specific ~16kDa band when stained with Trem2 antibody, not present in media conditioned from untransfected cells. This band corresponds to the same band seen in soluble fractions from total rat brain and media conditioned by primary rat microglia. In addition, this band is ~8kDa smaller than the full-length form of Trem2 detected in microglia, consistent with its identification as soluble Trem2.

**
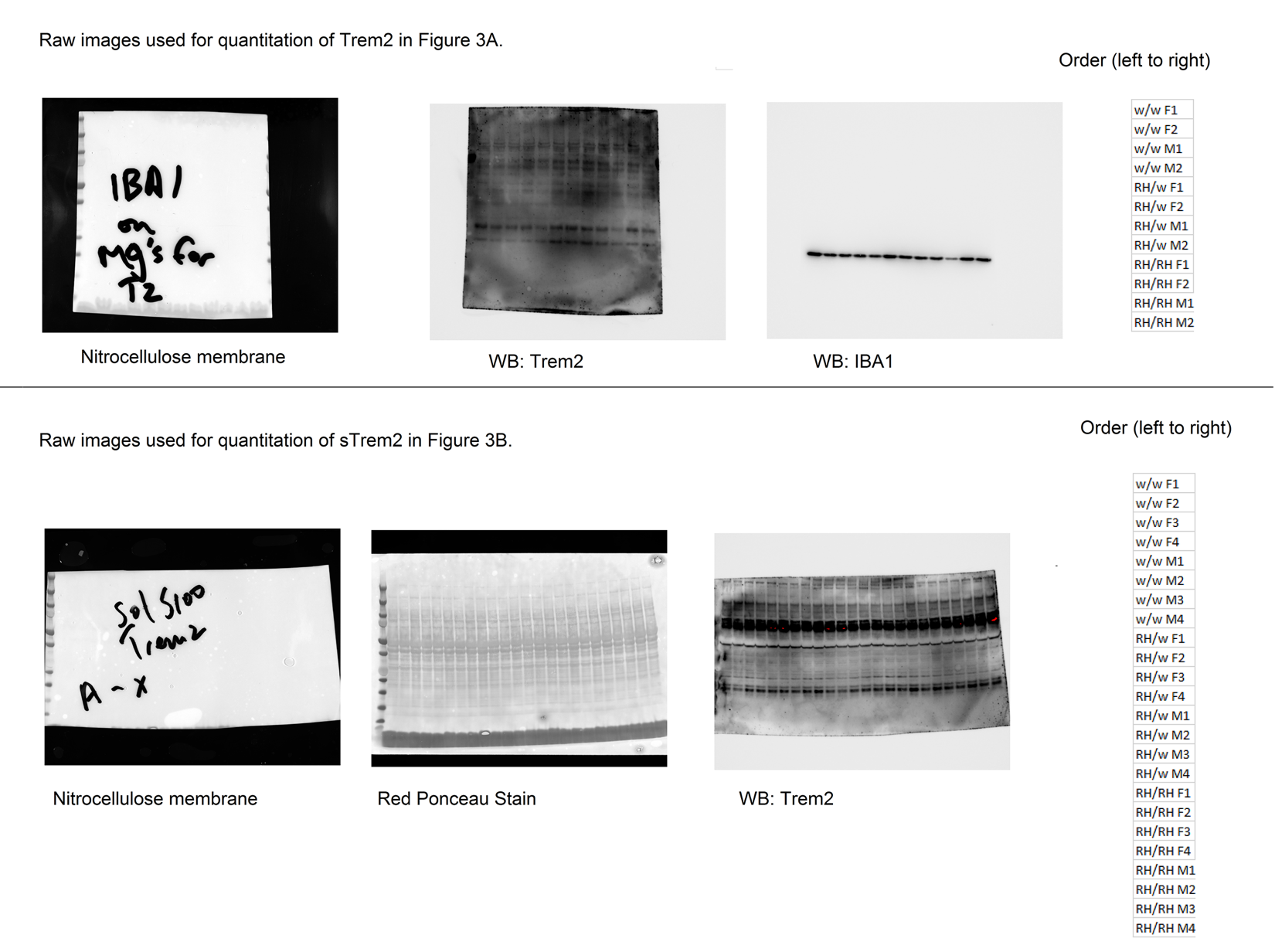
Figure S3. Raw blots used for quantitation shown in Fig. 3.**

**
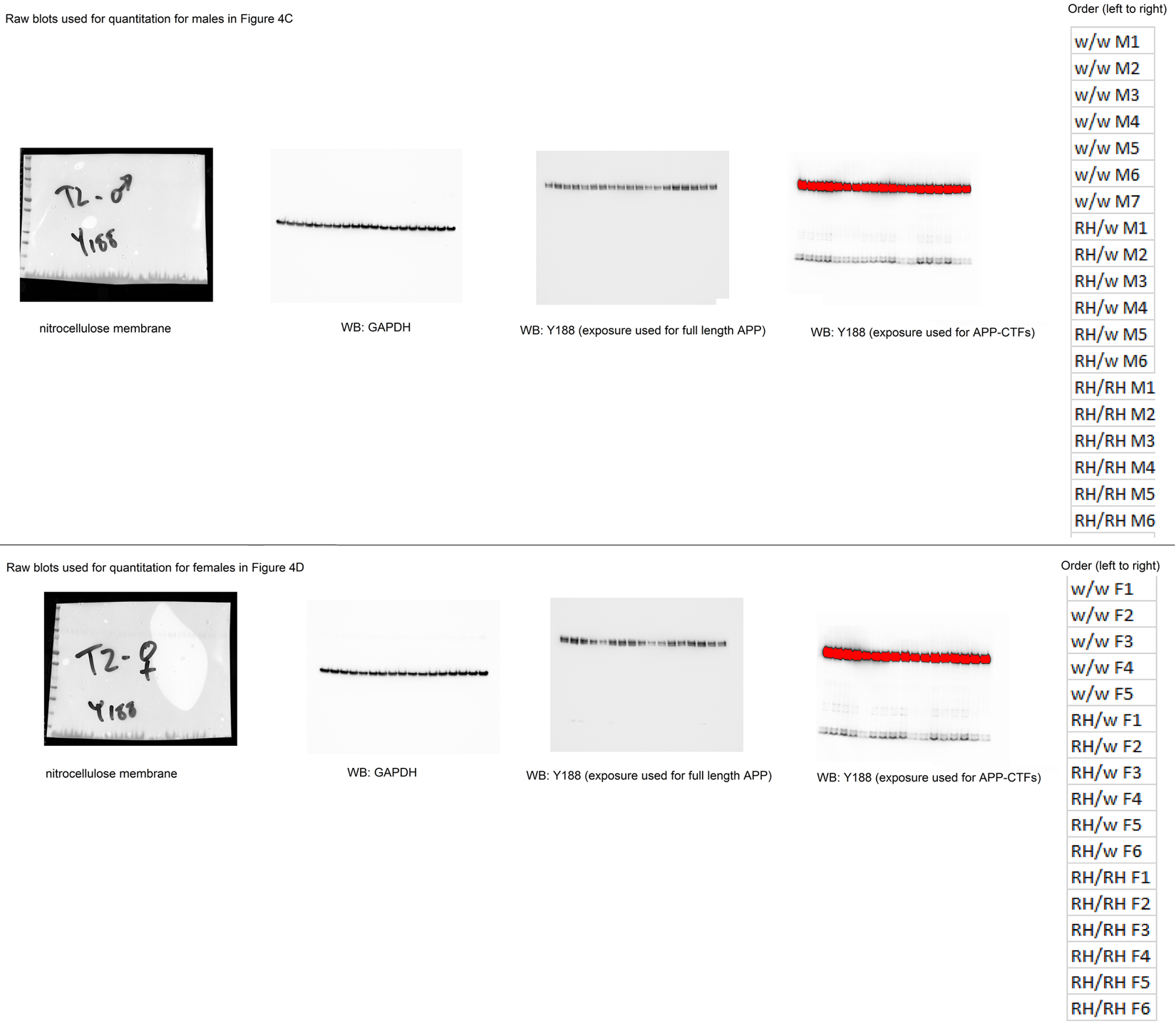
Figure S4. Raw blots used for quantitation shown in Fig. 4.**
